# Modelling Adaptation to Directional Motion Using the Adelson-Bergen Energy Sensor

**DOI:** 10.1371/journal.pone.0059298

**Published:** 2013-03-15

**Authors:** Andrea Pavan, Adriano Contillo, George Mather

**Affiliations:** 1 SISSA, Trieste, Italy; 2 Institut für Psychologie, Universität Regensburg, Regensburg, Germany; 3 PRISMA Cluster of Excellence & Institute of Physics (THEP), University of Mainz, Mainz, Germany; 4 School of Psychology, University of Lincoln, Lincoln, United Kingdom; Brain and Spine Institute (ICM), France

## Abstract

The motion energy sensor has been shown to account for a wide range of physiological and psychophysical results in motion detection and discrimination studies. It has become established as the standard computational model for retinal movement sensing in the human visual system. Adaptation effects have been extensively studied in the psychophysical literature on motion perception, and play a crucial role in theoretical debates, but the current implementation of the energy sensor does not provide directly for modelling adaptation-induced changes in output. We describe an extension of the model to incorporate changes in output due to adaptation. The extended model first computes a space-time representation of the output to a given stimulus, and then a *RC* gain-control circuit (“leaky integrator”) is applied to the time-dependent output. The output of the extended model shows effects which mirror those observed in psychophysical studies of motion adaptation: a decline in sensor output during stimulation, and changes in the relative of outputs of different sensors following this adaptation.

## Introduction

The motion energy model [1] has become established as the standard computational model for low-level motion sensing in the human visual system. In its original form it is a multistage model that includes four spatiotemporal filters (two for rightwards motion, and two for leftwards motion) oriented in space-time. These filters are created by combining pairs of spatial and temporal filters which are shifted in their spatial or temporal phase. The output of the spatiotemporal filters is squared before opponent energy is computed as the difference between left and right sensor outputs (i.e., *E_L_ – E_R_*). Physiological studies [2] have shown that the properties of each stage of the model correspond to the behaviour of cells in the Lateral Geniculate Nucleus (LGN), striate cortex (V1), and extrastriate cortex (V5/MT). De Valois et al. [2] found two sub-populations of non-directional V1 cells, one with a slow monophasic temporal response, and one with a fast biphasic temporal response. Moreover, these two populations are in approximate temporal quadrature and differ in the spatial phase of their receptive fields (RF). The same authors also reported that the RFs of some directional V1 cells can be constructed by a linear combination of fast biphasic and slow monophasic cells. Fast biphasic cells receive input from magnocellular cells in the LGN, whereas slow monophasic cells receive input from parvocellular cells in the LGN. Thus, directional V1 cells could receive the approximately temporal and spatial quadrature inputs required for motion detection by combining signals from the two non-directional sub populations which have their origins in magno and parvo cells of the LGN. In addition, there is physiological evidence that motion area V5/MT is the principal cortical area involved in motion opponency, corresponding to the model stage at which opponent energy is computed [3]–[6]. Area V5/MT has been found to contain mutually suppressive neural populations sensitive to motion in opposite directions, whereas in the primary visual cortex there is little evidence for motion opponency [3]. Georgeson and Scott-Samuel [7] added a normalization stage to the model, in which opponent energy is normalised with *flicker energy* (i.e., the sum of the directional motion energies: *E_L_ + E_R_*), because they found that opponent energy was a poor predictor of psychophysical direction discrimination performance. Normalised energy called *motion contrast* (*E_L_* – *E_R_*)/(*E_L_ + E_R_*) uses divisive inhibition to implement contrast gain control in the model, as suggested by Heeger [8]. Motion contrast was found to be a good predictor of direction discrimination performance over a wide range of contrast levels. Although this updated version of the motion energy model provides a good account of a wide range of psychophysical tasks, such as direction discrimination and lateral masking [9], [10] a crucial limitation of the model in its current form is that it cannot account for the well-known and dramatic effects of prolonged exposure to unidirectional motion (i.e., motion adaptation), such as the motion after-effect (MAE). These effects have played a pivotal role in both empirical and theoretical studies of motion perception for nearly 150 years [11], [12], so their exclusion from the energy model is a major limitation of the dominant theoretical scheme (previous attempts to apply the model to MAE data [15] have simply used the magnitude of sensor output during adaptation as a proxy for the strength of the resulting adaptation).

To address this limitation, the present study extends the motion energy model by introducing an additional stage in the form of a *RC* automatic gain-control circuit operating in time domain. The function of this stage is to regulate the gain of each motion sensor based on its recent exposure to directional motion. The performance of the extended model is tested by comparing its output with psychophysical data from the standard MAE obtained using stationary test patterns. Model output shows effects which mirror those observed in psychophysical studies of motion adaptation.

## Method

### 1. Computational Modelling

#### 1.1. RC Integrator

The new extension of the model implements divisive feed-forward gain control in motion sensors using a ‘leaky integrator’ circuit. A general feature is that the output signal at any point in time is a fraction of the input, proportional to the magnitude of the input in the past. The simplest form of leaky integrator circuit is known as an *RC* integrator. It is made up by a *resistor R* and a *capacitor C*. Applying a constant voltage *V_in_* to the input causes the potential difference across the resistor to follow an exponential function:



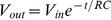
(Eq.1)


**Figure 1:**
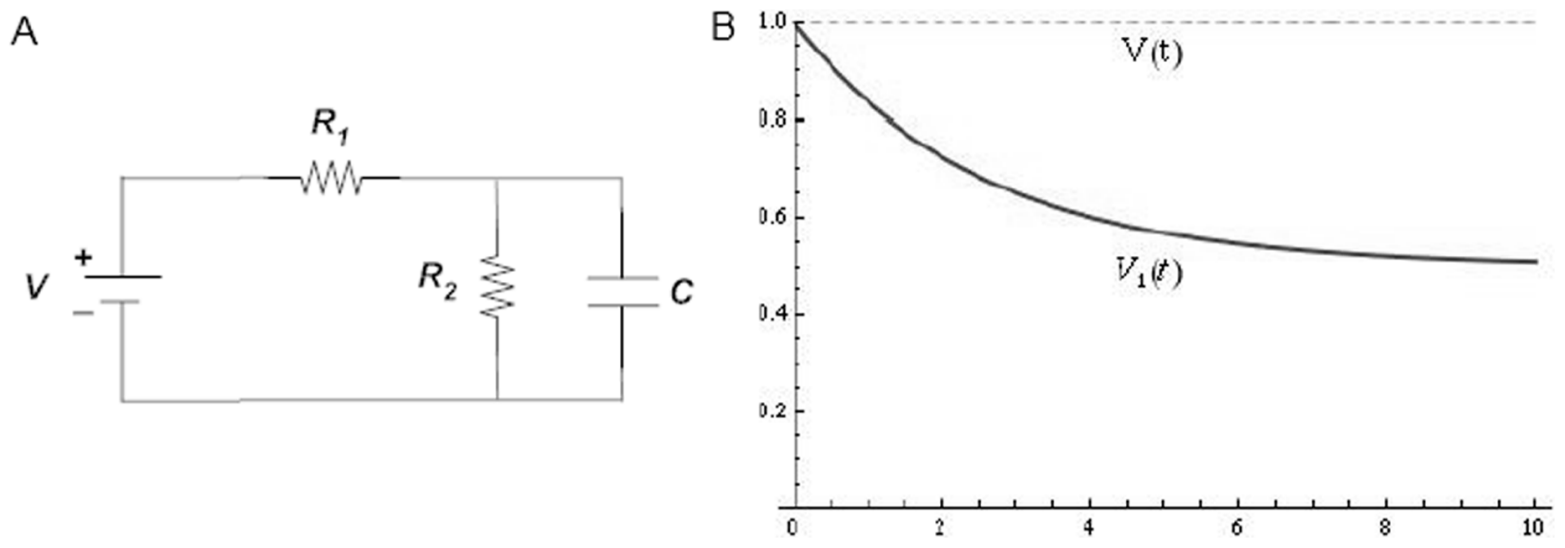
Schematic representation of the *RC* circuit (A) and its output (B). (A) The input signal is represented by the voltage generator *V*, while the output is the voltage difference across the resistor *R_1_*. (B) The dashed line indicates the input signal [*V(t)*], whereas the solid line indicates the output of the *RC* circuit [*V_1_(t)*]. The asymptotic behavior of the output is clearly visible at the far right of the plot.



Output tends asymptotically towards zero, at a rate that increases as the input value increases. This happens because there is a continuous storage of energy inside the capacitor, reducing the gain of the circuit (amount of current flowing through the resistor). This type of *RC* integrator is the same as that used by van de Grind et al. [13], where the reduced gain was defined as *adaptation*. However, in such a simple *RC* integrator the output has a limiting value at zero. This means that a specific neural circuit will reach zero efficiency if subjected to a stimulus for a sufficiently long amount of time. Consequently exposure to a constant directional stimulus would cause the corresponding sensor to become completely silent. This behaviour is very rarely observed physiologically or psychophysically. van de Grind et al. [13] fixed this issue by rescaling the adaptation using an *ad hoc* factor that, if chosen carefully as much smaller than 1, avoids complete suppression. In the present study we propose an alternative and more efficient solution, and we embed the solution in a full implementation of the motion sensor. Specifically, we added another resistor to the *RC* integrator, as shown in Figure 1 (panel A). The additional branch allows a portion of the current to avoid the capacitor and to flow directly through the two resistors, shifting the asymptotic value of the output from zero to a positive quantity: *V_asym_*  =  *aV_in_*, where:

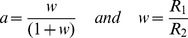



That is, the ratio *w* between the two resistors defines the portion *a* of signal that keeps flowing indefinitely in the grid (Figure 1 – Panel B). From a biological perspective, we constrained the motion response to converge on a fixed ratio of the original response, whose precise value can be established empirically with a psychophysical experiment. Thus, the channel described by the modified integrator can only be adapted up to a certain level, indefinitely maintaining a certain amount of “sensitivity”.

Moreover, it should be noted that linearity between input and output is also conserved. For example, given a second input *V'_in_  =  2V_in_* the new asymptotic output is given by:



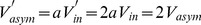
(Eq.2)


In addition, it is worth focusing on some particular relationships between the two resistors *R_1_* and *R_2_*:

(i) *R_2_* » *R_1_*: the second resistor has an extremely high value, which practically isolates the additional branch of the circuit, bringing us back to the simple *RC* integrator. In this limit *w* is extremely small, and so is the ratio *a*, which corresponds to the above described situation of complete saturation (i.e., *V_asym_*  =  0).

(ii) *R*_2_  =  *R*_1_: the two resistors have exactly the same value. This gives *w* = 1, and consequently *a* = 1/2, which corresponds to a 50% saturation (i.e., *V_asym_*  =  *V_in_*/2).

(iii) *R*_2_ « *R*_1_: the second resistor offers almost no resistance to the current flow, thus excluding the branch with the capacitor. In this limit *w* has a very large value, while the ratio *a* tends to unity, which corresponds to the situation of no saturation (i.e., *V_asym_*  =  *V_in_*).

The observed ratio (*a_obs_*) will lie somewhere in the interval [0, 1] and, as already stated, must be estimated from experimental measurements. The other parameter of great importance in the model, and that has to be derived from experiments, is the *decay time*, that is, *τ*  =  *R_1_C*, measuring the amount of time needed by the motion sensor to lose most of its gain and approach its asymptotic value.

It should be noted that all the arguments expressed above were based on the assumption of a constant input stimulus. Nonetheless it is necessary to remark that we are able to derive the response of our modified *RC* integrator to a stimulus that is a generic function of time. Making use of the redefinitions *V_in_*  =  *z* and *V_out_*  =  *y*, such a response is given by:



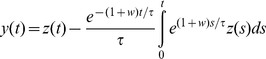
(Eq.3)


Equation 3 contains an integral of the input over the previous time intervals, encapsulating the required feature of gain control based on the value of the input in the past.

The effect decays as the input signal *z(t)* ceases, so the MAE is expected to decay after the end of the adapting stimulus, *t_A_*. This would be in agreement with previous psychophysical observations on the MAE [11], [12], [14]. In the next section the modified *RC* integrator will be embedded in the motion energy model, after which its output will be compared to published psychophysical data.

#### 1.2. The Extended Motion Energy Model

**Figure 2:**
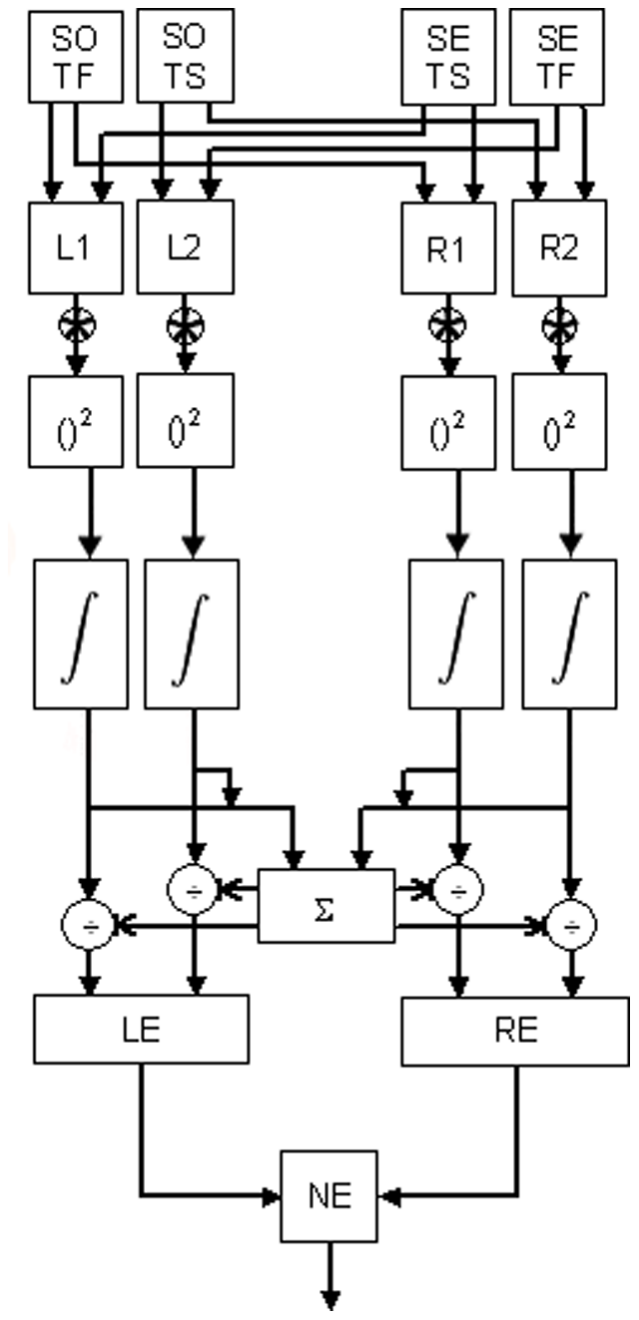
The extended motion energy model. The integrator stage is located after the squaring stage. See text for details.



The extended model is outlined in Figure 2 and was implemented in Matlab. The spatial and temporal profiles of the filters of the model covered 2.25 deg of space and 1 s of time. Spatial filter profiles were even (*EV*) and odd (*OD*) Gabor functions of the form:

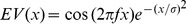
(Eq.4)




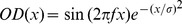
(Eq.5)


where ƒ is 1.95 cpd and σ is 0.28 deg. Temporal filters had the following form, taken from Adelson and Bergen [1] Eq. (6):



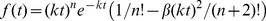
(Eq.6)


The value of *k* scales the response into time units and was set to 100, while *n* sets the vertical width of the filter [15]. The parameter *n* was equal to 9 for the slow temporal filter and 6 for the fast temporal filter, as used in previous modelling [16]–[20]. The parameter *β* reflects the weighting of the negative phase of the temporal impulse response relative to the first positive phase and was set to 0.9 [17], [18], [21]. The product of the even and odd spatial profiles [i.e., *EV(x)* and *OD(x)*] with the two temporal profiles ƒ_slow_(t) and ƒ_fast_(t)] creates four (space-time) separable filters (first layer of the model; Figure 2). These filters were combined to obtain in turn four sensors oriented in space-time; two oriented for leftward motion and two for rightward motion (second layer of the model; Figure 2). The two members of each pair are approximately 90 deg out of phase with each other [1]. Convolving these four filters with the same input image gives four response matrices that are subsequently squared (third layer of the model; Figure 2). We label the matrices resulting from this squaring as *R_L1_*_,_ *R_L2_*, *R_R1_*, and *R_R2_*.

We then implemented the adaptation stage by introducing the modified *RC* integrator (fourth layer of the model; Figure 2). That is, at each time slice (row in the output matrix), the output of each convolution stage was multiplied by a factor:



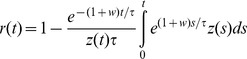
(Eq.7)


where *z(t)* is the output of the respective sensor averaged over the whole spatial range as a function of time in the recent past. For example, *r_L1_(t)* will be obtained taking as *z(t)* the spatial average of *R_L1_(x,t)*. Notice that the above formula directly derives from Eq. 3. Formally this can be written as follows:



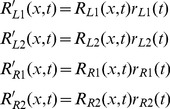
(Eqs.8)


Then, as required in the standard model, we summed the responses derived from the two pairs of filters to compute leftward and rightward motion energies. The output matrices are respectively defined as:



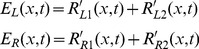
(Eqs.9)


Opponent energy is then computed using the following measure of net Energy:



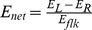
(Eq.10)


with a normalization factor, called *flicker energy* [7], defined as an average over the whole output matrix:



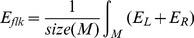
(Eq.11)


### 2. Psychophysics

The output of the extended motion energy model was fitted to MAE data reported in Experiment 1 of Hershenson [14]. Briefly, the stimulus was a horizontal squarewave grating that moved upward with a temporal and spatial frequency of 6 Hz and 2.5 c/deg, respectively (velocity  =  2.4 deg/s).

Observers rated the strength of the MAE (approximately every 1 s) during the test period that was presented immediately after the cessation of the adapting motion. Observers used an 11-point scale in which 10 represented the strength of the perceived motion of the MAE immediately upon cessation of the motion of the adapting stimulus (i.e., its initial perceived strength), and 0 represented no perceptible motion. For the purposes of comparison with the model output we shall present the data obtained by Hershenson [14] at adapting durations of 120 and 150 s.

**Figure 3:**
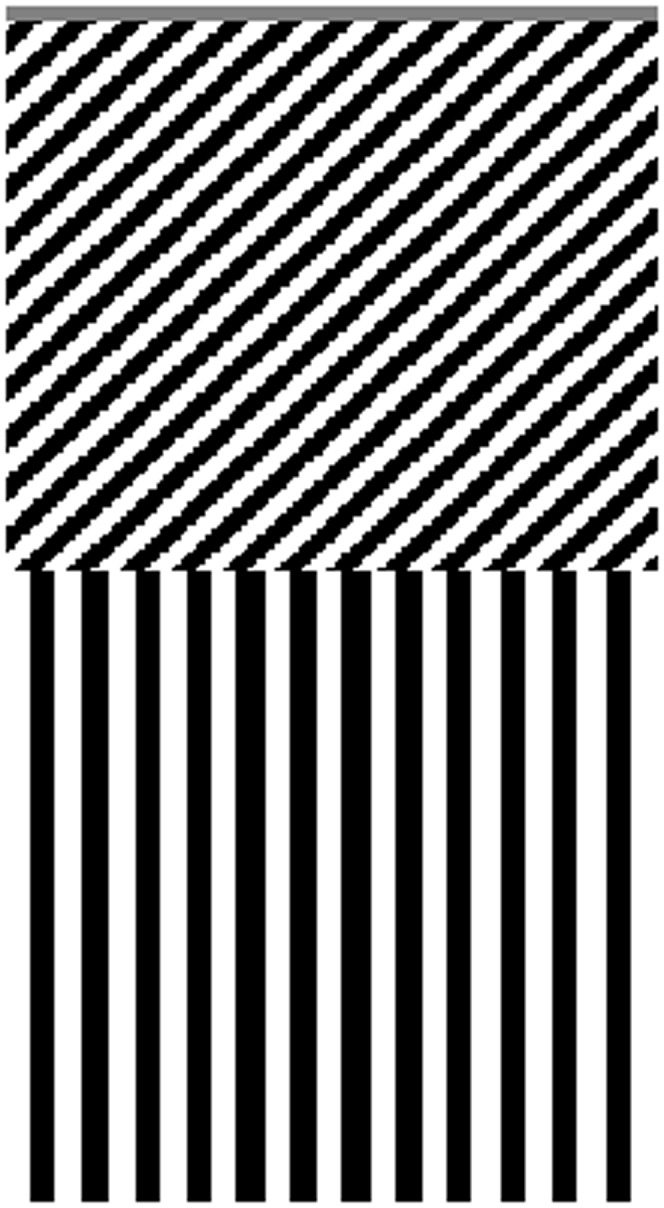
Space-time (xt) representation of the stimulus. The input stimulus consisted of a space-time representation of a squarewave adapting grating drifting leftward and with duration of 120 or 150 s (the image represents an adapting stimulus of 120 s). The adapting stimulus was immediately followed by a stationary test stimulus. The space-time presentation of the adapting pattern (i.e., tilted bars) appears quite coarse, but this is a merely graphic artifact caused by the necessity to resize an image with a vertical dimension much larger than the horizontal one. However, the input matrix is composed of smoothly leftward drifting black and white bars.

The spatiotemporal characteristics (drift velocity) of the input stimulus in the model matched those of the stimulus reported in [14]. The input stimulus was encoded in space-time (i.e., *xt*) with a spatial dimension covering 4.5 deg sampled at intervals of 0.028 deg, and a temporal dimension of 260 s sampled at intervals of 10 ms. In particular, stimuli consisted of a spatiotemporal representation of a leftward drifting squarewave grating (adapting pattern) and a stationary test grating (Figure 3). Adapting stimuli consisted of 120 or 150 s of unidirectional drift, within a grey matrix.

## Results

**Figure 4:**
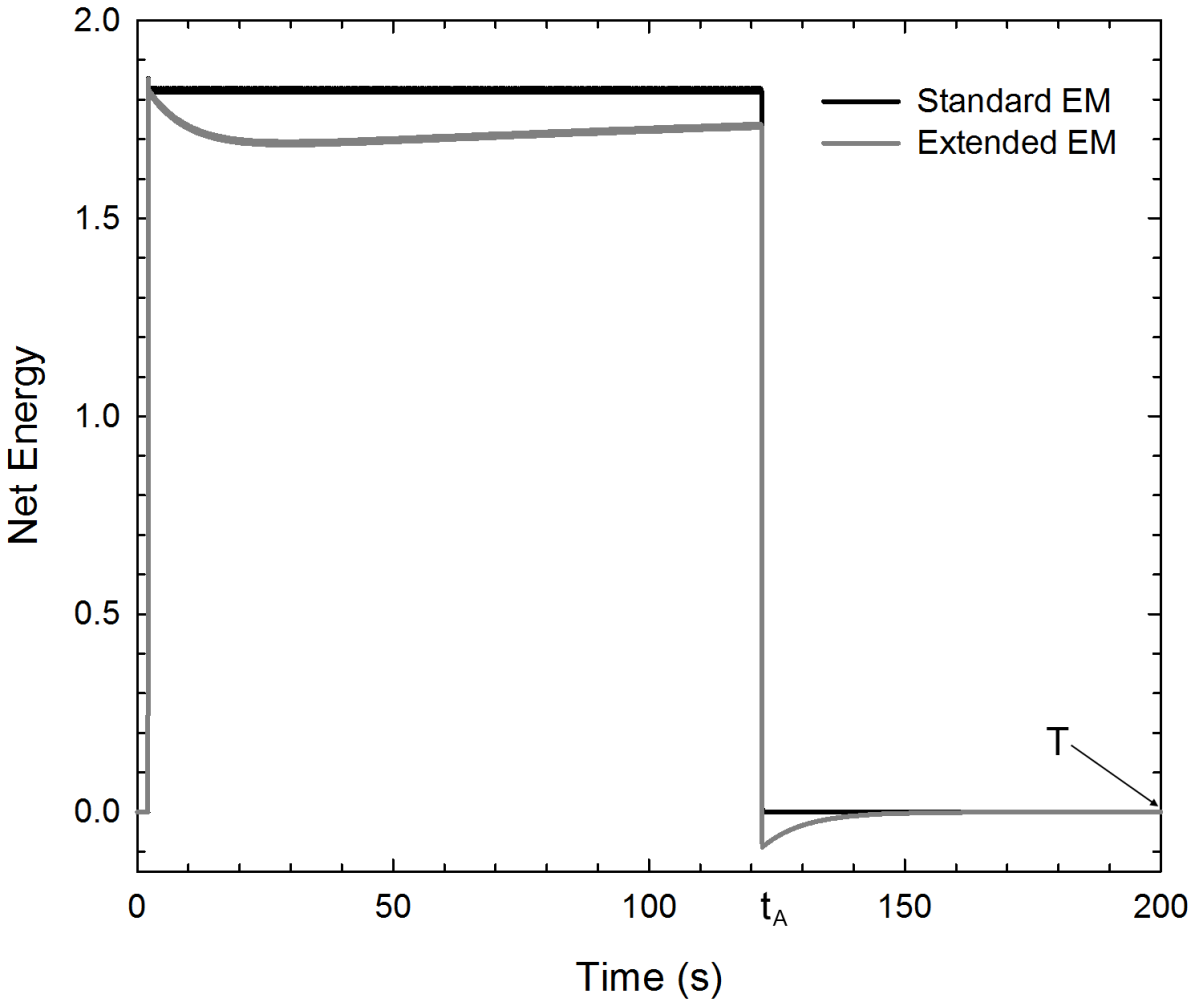
Comparison between the outputs of the standard (black line) and extended (gray line) motion energy models. The outputs are averaged across the spatial dimension. The dashed line indicates no motion. *t_A_* indicates the offset of the adapting pattern and *T* the duration of the stimulus.

We first compared the time-dependent output of the standard energy model (i.e., lacking the integrator) against the output of the extended model. The stimulus contained directional motion for the first 120 seconds, followed by a stationary test pattern for the remaining stimulus period. The output of the models is shown in Figure 4; combining the four filters in the way described above results in a net energy value that, when averaged over the spatial dimension, can be visualised as a function of time. As can be seen in the Figure, the standard model maintains a constant rightward output during adaptation, and a constant non-directional output during the test interval. The extended model, on the other hand, shows an initial drop in directional energy at the start of the adapting period, and an ‘after-effect’ lasting approximately 20 seconds at the cessation of adaptation; net energy signalled by the sensors is in the opposite direction to the adapting stimulus.

In the section ‘*RC Integrator*’ we stated that the two parameters of the leaky integrator, *a* and *τ*, must be inferred from experimental measurements. This was achieved as follows. For the sake of simplicity we performed this part of the analysis using ready-made time functions as input stimuli *z_L_* = *z_L_(t)* and *z_R_* = *z_R_(t)* averaged over the spatial dimension. We assume that a unidirectional adapting stimulus is applied for duration of *t_A_* in one specific direction followed by a static test stimulus. The motion sensor coding for the adapting direction will thus be adapted from the beginning of the adapting period, while the motion sensor coding for the opposite direction will arrive at *t_A_* with no prior adaptation. This will cause an imbalanced response to test stimulus, which can be quantified in terms of *net energy*. Again for the sake of simplicity we will consider this function as already averaged over the spatial dimension, allowing us to make use of the following



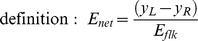
(Eq.12)


where *y_L_ = y_L_(t)* and *y_R_ = y_R_(t)* are the spatially averaged output of the left and right channel and the *flicker energy* is now defined as an average over the duration (*T*) of the stimulus (as the spatial average is already implied in the definition of *E_net_*, so that the definition actually coincides with the one of Eq. 11)



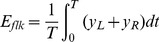
(Eq.13)


*E_net_* ranges between some negative number *-N* (when *y_L_* vanishes) and *+N* (when *y_R_* vanishes). Such a function can be considered as an index of directionality of the motion response: positive (negative) means left- (right-) ward, while zero means total ambiguity. As shown in Figure 4, immediately after the offset of the adapting pattern (*t_A_*) the model signals motion in the opposite direction to that of the adapting pattern. The effect exponentially decays as time increases, so the MAE is expected to decay soon after the onset of the test stimulus.

To find the best-fitting values of *a* and *τ*, we define the inputs



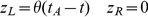
(Eq.14)


written in terms of the step function *θ*(*q*), equal to 1 when *q>0* and 0 otherwise. Making use of the definition Eq. 12, we defined a variable *p* as the ratio between the net energy evaluated at a time-point of the test stimulus at which we wish to measure the model output (i.e., *t_elapsed_*) and its *minimum* value *E*_min_ = *E*_net_ *(t_A_)*



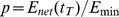
(Eq.15)


being *t_T_* = *t_A_+t_elapsed_*, so that it will assume the maximum value, *p*  =  1, when evaluated at *t_T_* ≡ *t_A_*. The function *p* can be calculated analytically with respect to the input functions of Eq. 7, giving the expression



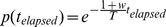
(Eq.16)


that only depends on the time-point on the test pattern at which the output model is evaluated. The estimation of *τ* and *w* (from which *a* can be inferred) was obtained by fitting Eq. 16 to Hershenson’s data. Clearly the comparison is based on the assumption that the observer’s ratings of MAE are directly proportional to the model output *E_net_*. In order to compare the data with the output of our model, they were exponentiated to base 10 and then divided by 10, in such a way as to span the interval [0, 1]. The estimated values were: *a*  =  0.911 and *τ*  =  95.60 s. Using these parameters in the extended Adelson-Bergen energy model, we simulated a set of outputs corresponding to the selected data (*t_A_* = {120 s, 150 s} and *t_elapsed_* = [0 s, 15 s]), then calculated the RMSE separately for each adaptation duration. Figure 5 shows the comparisons between psychophysical results of Hershenson [14] and the predictions of the extended motion sensor model, for 120 and 150 s adapting durations. Data and model clearly show that MAE strength decays exponentially as the test period progresses. It is not necessary to plot predictions for the standard model, since it would simply predict no MAE at any time (a horizontal line at zero). The extended motion energy model accurately fits the exponential decay of the adaptation; the RMSE we obtained were 0.037 and 0.059 for 120 and 150 s adaptations, respectively.

**Figure:**
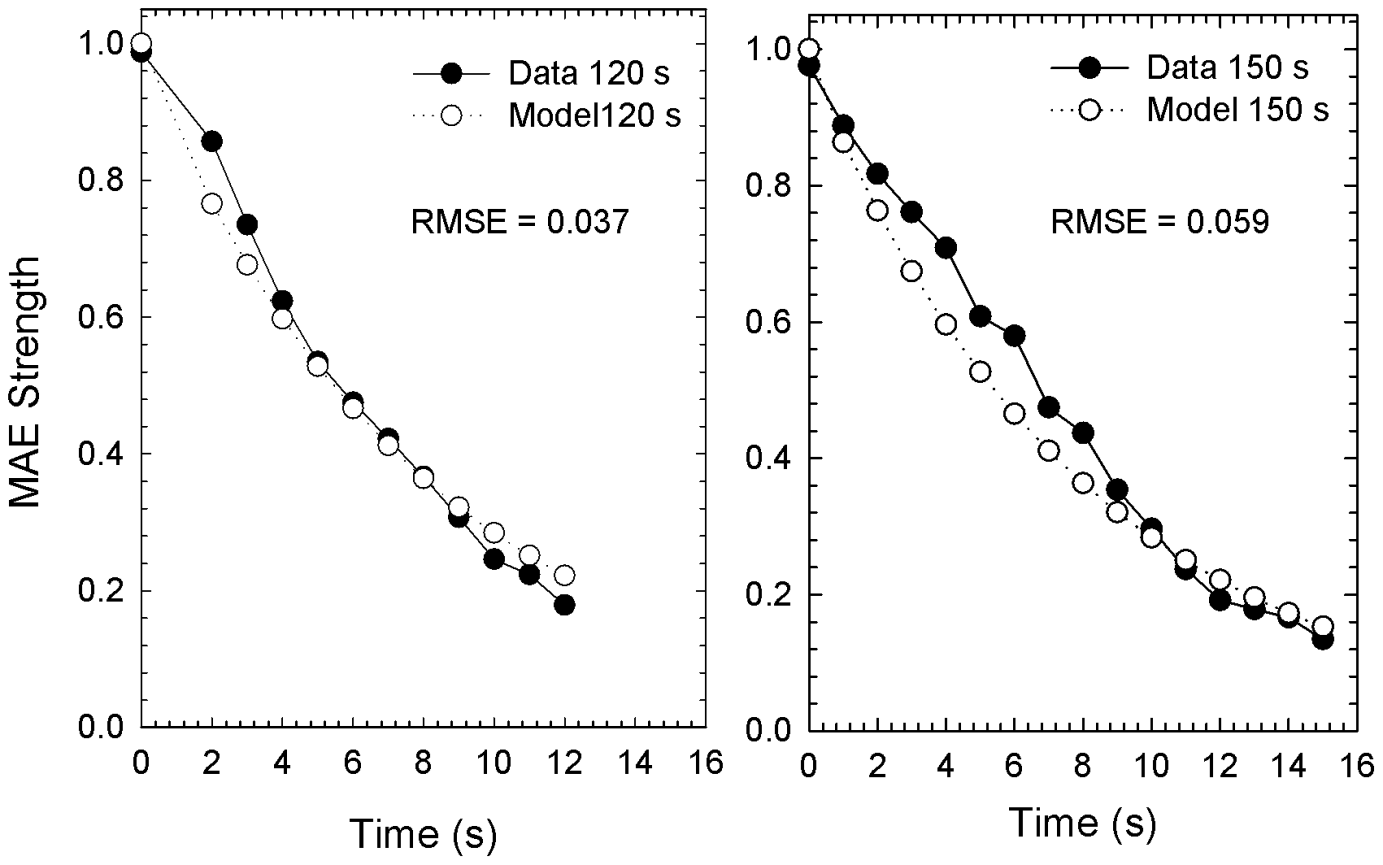
Extended motion energy model output fitted to the psychophysical data. MAE strength is shown as a function of the time (s) on the test phase at which the model output is evaluated (filled circles) [14]. Model fits are shown in separate panels for two adaptation durations: 120 s (left panel) and 150 s (right panel). The pair of *a* and *τ* that produced the best fit were *a*  =  0.911 and *τ*  =  95.60. The RMSE obtained for 120 s adaptation was 0.037, whereas for 150 s adaptation was 0.059.

## Discussion

Computational results show that the output of the extended energy model is able accurately to account for the psychophysical adaptation data obtained in the MAE study of Hershenson [14]. In particular, the extended Adelson-Bergen model can predict the exponential decay of the MAE as the test interval increases.

*RC* gain-control circuits of the kind used here have a long history in the context of visual processing. They have been used in both physiological studies [21] and in psychophysical studies [22]. This paper represents the first attempt to employ such a circuit in a plausible computational model of human motion detection. Exponential decay is a characteristic feature of leaky integrators, and has also been noted as a psychophysical property of the decay in MAE adaptation [22], [23], and as an electrophysiological property of adaptation in cortical direction-selective neurons [24], [25]. There is psychophysical and physiological evidence for multiple sites of adaptation and corresponding multiple decay time constants [26]. In this regard it should be noted that Hershenson’s data also show an increase in MAE strength as adaptation duration increases from 30 to 180 s, which we have not observed in the behaviour of the model. This failure may reflect the fact that MAEs are due to combination of multiple adaptation processes at different neural sites, with different time constants, and the model incorporates only one such process. Thus it seems that, as well as the gain-control circuit which is modelled here, other gain-control circuits are likely to be present in the motion pathway.

All electrical circuits, whether metallic or neural, have resistance and capacitance, so time-dependent behaviour of the kind modelled in this paper is likely to ubiquitous. However, current theories of adaptation argue that it is not simply a by-product of resistance and capacitance, but is an adaptive feature of neural processing which serves to reduce redundancy, conserve energy, and maximise processing efficiency [11].
